# Evaluation of RESPOND, a patient-centred program to prevent falls in older people presenting to the emergency department with a fall: A randomised controlled trial

**DOI:** 10.1371/journal.pmed.1002807

**Published:** 2019-05-24

**Authors:** Anna Barker, Peter Cameron, Leon Flicker, Glenn Arendts, Caroline Brand, Christopher Etherton-Beer, Andrew Forbes, Terry Haines, Anne-Marie Hill, Peter Hunter, Judy Lowthian, Samuel R. Nyman, Julie Redfern, De Villiers Smit, Nicholas Waldron, Eileen Boyle, Ellen MacDonald, Darshini Ayton, Renata Morello, Keith Hill

**Affiliations:** 1 School of Public Health and Preventive Medicine, Monash University, Melbourne, Victoria, Australia; 2 Alfred Health, Melbourne, Victoria, Australia; 3 School of Medicine, University of Western Australia, Perth, Western Australia, Australia; 4 Department of Geriatric Medicine, Royal Perth Hospital, Perth, Western Australia, Australia; 5 Harry Perkins Institute for Medical Research, Perth, Western Australia, Australia; 6 Melbourne EpiCentre, University of Melbourne, Melbourne, Victoria, Australia; 7 Melbourne EpiCentre, Melbourne Health, Melbourne, Victoria, Australia; 8 School of Primary and Allied Health Care, Monash University, Melbourne, Victoria, Australia; 9 Allied Health Research Unit, Monash Health, Melbourne, Victoria, Australia; 10 School of Physiotherapy and Exercise Science, Curtin University, Perth, Western Australia, Australia; 11 Bolton Clarke Research Institute, Bolton Clarke, Melbourne, Victoria, Australia; 12 Department of Psychology and Ageing & Dementia Research Centre, Faculty of Science and Technology, Bournemouth University, Poole, United Kingdom; 13 Westmead Applied Research Centre, University of Sydney, Westmead, New South Wales, Australia; 14 Health Networks Branch, System Policy and Planning, Department of Health, Government of Western Australia, Perth, Western Australia, Australia; 15 Emergency Department, Royal Perth Hospital, Perth, Western Australia, Australia; University of Cambridge, UNITED KINGDOM

## Abstract

**Background:**

Falls are a leading reason for older people presenting to the emergency department (ED), and many experience further falls. Little evidence exists to guide secondary prevention in this population. This randomised controlled trial (RCT) investigated whether a 6-month telephone-based patient-centred program—RESPOND—had an effect on falls and fall injuries in older people presenting to the ED after a fall.

**Methods and findings:**

Community-dwelling people aged 60–90 years presenting to the ED with a fall and planned for discharge home within 72 hours were recruited from two EDs in Australia. Participants were enrolled if they could walk without hands-on assistance, use a telephone, and were free of cognitive impairment (Mini-Mental State Examination > 23). Recruitment occurred between 1 April 2014 and 29 June 2015. Participants were randomised to receive either RESPOND (intervention) or usual care (control). RESPOND comprised (1) home-based risk assessment; (2) 6 months telephone-based education, coaching, goal setting, and support for evidence-based risk factor management; and (3) linkages to existing services. Primary outcomes were falls and fall injuries in the 12-month follow-up. Secondary outcomes included ED presentations, hospital admissions, fractures, death, falls risk, falls efficacy, and quality of life. Assessors blind to group allocation collected outcome data via postal calendars, telephone follow-up, and hospital records. There were 430 people in the primary outcome analysis—217 randomised to RESPOND and 213 to control. The mean age of participants was 73 years; 55% were female. Falls per person-year were 1.15 in the RESPOND group and 1.83 in the control (incidence rate ratio [IRR] 0.65 [95% CI 0.43–0.99]; *P* = 0.042). There was no significant difference in fall injuries (IRR 0.81 [0.51–1.29]; *P* = 0.374). The rate of fractures was significantly lower in the RESPOND group compared with the control (0.05 versus 0.12; IRR 0.37 [95% CI 0.15–0.91]; *P* = 0.03), but there were no significant differences in other secondary outcomes between groups: ED presentations, hospitalisations or falls risk, falls efficacy, and quality of life. There were two deaths in the RESPOND group and one in the control group. No adverse events or unintended harm were reported. Limitations of this study were the high number of dropouts (*n* = 93); possible underreporting of falls, fall injuries, and hospitalisations across both groups; and the relatively small number of fracture events.

**Conclusions:**

In this study, providing a telephone-based, patient-centred falls prevention program reduced falls but not fall injuries, in older people presenting to the ED with a fall. Among secondary outcomes, only fractures reduced. Adopting patient-centred strategies into routine clinical practice for falls prevention could offer an opportunity to improve outcomes and reduce falls in patients attending the ED.

**Trial registration:**

Australian New Zealand Clinical Trials Registry (ACTRN12614000336684).

## Abstract 2

Darshini Ayton and colleagues report on a fall prevention programme for people discharged from the emergency department post-fall, evaluated in a randomised controlled trial.

## Author summary

**Why was this study done?:**

- Falls are a leading reason that older adults present to emergency departments (EDs).
- There is systematic review evidence for interventions to reduce falls in older people living in the community.
- When similar interventions are applied to those presenting to the ED with a fall, there is a lack of effectiveness.

**What did the researchers do and find?:**

- A randomised-controlled trial was undertaken to investigate whether a 6-month telephone-based patient-centred program—RESPOND—reduced falls and fall injuries in older people presenting to the ED after a fall.
- RESPOND is a patient-centred falls prevention program developed specifically for community-dwelling older people presenting to the ED with a fall. It consists of a home-based risk assessment; 6 months telephone-based education, coaching, goal setting, and support for evidence-based risk factor management; and linkages to existing services.
- RESPOND had an effect on falls and fractures but not fall injuries.

**What do these findings mean?:**

- Our study provides evidence for a telephone-based, patient-centred falls prevention program to reduce falls and fractures in older people presenting to the ED with a fall.
- This approach may empower and support older people to participate in falls prevention activities.

## Introduction

The growing number of emergency department (ED) presentations by older people is a challenge to healthcare services worldwide [1–3]. Falls are the leading cause of ED presentations in people aged 60 years and older [4] and account for almost 50% of all incident injury presentations [5]. In the United Kingdom, an estimated 4 million emergency presentations for falls occur annually [6]. A recent United States study estimated that the number of fall-related injuries treated in the ED increased from 1.6 million in 2001 to 2.4 million in 2012, and this is projected to increase to 5.7 million by 2030 [7]. The cost of ED visits for nonfatal fall injuries in the US in 2012 was estimated to amount to US$8.2 billion [8]. Falls result from a combination of risk factors that relate to both the individual and the environment. Prevention programs aim to reduce, eliminate, or manage identified risk factors via multifactorial interventions [9]. There is good evidence for interventions to reduce falls in older people living in the community [10,11]. Despite this, when similar interventions are applied to those presenting to the ED with a fall, there is a lack of effectiveness [12–15]. This may be due to low levels of intervention prescription by ED staff and/or low uptake of interventions by older people. Only 3 in every 100 older patients presenting to the ED with a fall receive guideline care [16] and only 1 in 5 participants (21%) reported in a pooled analysis of RCTs had full adherence to prescribed home-based falls exercise programs [17]. The ED may be a challenging environment in which to deliver falls prevention interventions, as staff, workflows, and processes are focused on managing the acute care needs of a patient (e.g., injury assessment and management) as opposed to prevention. Older people who experience a fall that leads to an ED attendance are frailer, have multimorbidity, complex social issues, and more severe injuries when compared with those who do not attend the ED as a result of a fall [18,19]. These differences highlight that different falls prevention interventions may be needed to address the more complex ED population.

We developed a new intervention—RESPOND—with the aim of reducing falls in this patient group. RESPOND was specifically designed to provide personalised and timely education and support to improve knowledge, self-efficacy, and participation in evidence-based falls prevention activities [20]. The name RESPOND was coined based on an underlying philosophy of, ‘respond to the first fall to prevent the second’. The program also aimed to strengthen linkages between the ED and community care. The design of RESPOND was informed by the ‘Choice of Health Options In prevention of Cardiovascular Events (CHOICE)’ program, which successfully utilised a telephone-based motivational coaching approach to improve the modifiable risk profiles and risk factor knowledge of acute coronary syndrome survivors [21,22]. While targeted to a different clinical group (cardiac patients versus older people who have fallen), best-practice guidelines for both populations recommend risk factor assessment and management, which commonly relate to behavioural and lifestyle modification. RESPOND adopted the principles applied in the CHOICE intervention—an initial one-hour face-to-face session followed by telephone-based motivational coaching supported by education modules that included patient information leaflets. Differences between the two interventions were content (falls- versus cardiac-specific), duration (6 versus 3 months), and intensity (45- versus 10-minute phone calls). The aim of this RCT was to investigate the effectiveness of RESPOND for reducing falls and fall injuries in older people after presenting to the ED with a fall.

## Methods

### Ethics

Ethics approvals were obtained from Alfred Health (HREC 439/13) and Royal Perth Hospital (REG 13–128), Curtin University Human Research Ethics Committee (HR 43/2014), University of Western Australia Human Research Ethics Committee (RA/4/1/6692), and Monash University Human Research Ethics Committee (MUHREC CF13/3869-201300). Those eligible and agreeing to participate provided informed written consent before taking part in the trial.

### Study design and participants

A RCT was conducted in The Alfred and Royal Perth Hospitals in Australia. This trial was registered with the Australian New Zealand Clinical Trials Registry (ACTRN12614000336684) and the protocol has been published elsewhere [20]. The CONSORT checklist is provided as S1 CONSORT checklist.

People aged 60–90 years who attended the ED as a result of a fall between 1 April 2014 and 29 June 2015 were considered for recruitment. RESPOND sought to recruit people who were discharged directly home from the ED or who had a short inpatient stay, on the basis that these people would be least likely to receive comprehensive geriatric assessment and management, including falls risk assessment and management, and therefore remain at risk of further falls.

Inclusion criteria were a planned hospital stay (ED and/or hospital admission) of 72 hours or less. A fall was defined as ‘an event resulting in a person coming to rest inadvertently on the ground, floor, or other lower level’ [23]. Exclusion criteria were having planned discharge to residential aged care; receiving palliative care or presence of a terminal illness; requiring hands-on assistance to walk from another individual (people could use an assistive device such as a walker); being unable to use a telephone; being non-English speaking; the presence of cognitive impairment (Mini Mental State Examination [MMSE] score <23) [24], social aggression, or a history of psychosis. People living further than 50 kilometres from trial sites were also excluded, as it was not feasible to perform the initial home risk assessment visit. Research staff screened patient records and interviewed patients in the ED on a daily basis to identify potential participants. Those eligible and agreeing to participate provided informed written consent before taking part in the trial.

### Randomisation and masking

Following recruitment, participants were randomly assigned (1:1) to one of two groups using a web-based system. The randomisation sequence applied permuted block randomisation (random blocks sizes of 2 and 4) stratified by recruitment site to ensure equal control and intervention participant numbers across sites. Group allocation was stored in a web-based database and was not revealed to staff or the participant until after completion of the baseline assessment to ensure the assessment was unbiased. Outcome assessors who collected data on the primary and secondary outcomes were blinded to group allocation, as was the statistician who conducted the outcome analysis.

### Procedures

**Fig 1:**
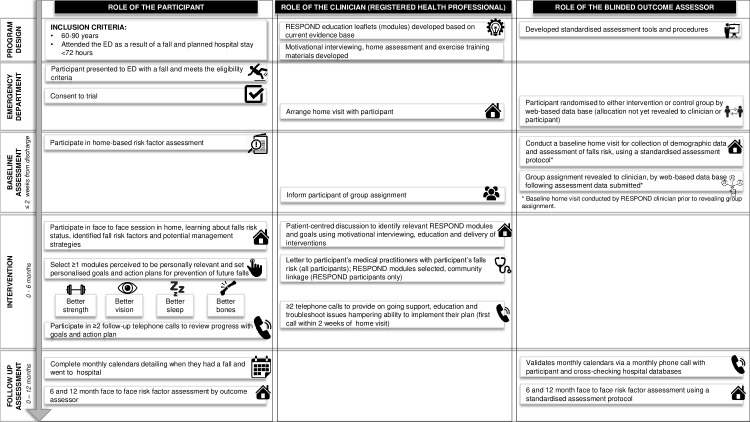
An overview of key study activities for RESPOND. ED, emergency department.

Fig 1 provides an overview of the key study activities. Following recruitment, a RESPOND clinician (registered healthcare professional) contacted participants to arrange a baseline home visit for collection of demographic data and assessment of falls risk. A falls risk factor assessment was completed by the RESPOND clinician using the validated Falls Risk for Older People in the Community (FROP-Com) tool [25,26]. Functional health literacy was measured using the Health Literacy Questionnaire (HLQ) [27]; health-related quality of life was assessed using the EuroQol five dimensions questionnaire (EQ-5D-5L) [28,29]. Falls self-efficacy was assessed using the Falls Efficacy Scale–International (Short version) (Short FES-I) [15]. The baseline assessment was undertaken using a standardised assessment protocol, and data were entered directly into a web-based database via an iPad. Following electronic submission of this assessment form, group allocation was revealed to the clinician, who informed the participant of their group assignment.

Both control and intervention groups continued with standard care as initiated by ED staff and their healthcare providers (e.g., investigations, multidisciplinary assessment, referral to specialists and falls prevention services, post-discharge nurse telephone contact). A letter was also sent by the research team to all participants’ usual care medical practitioners, informing them of the participant’s FROP-Com falls risk status (low, moderate, or high), and if they had scored ‘moderate or severe anxiety or depression’ on the EQ-5D. For participants assigned to the RESPOND intervention, the clinician extended the duration of the home visit to provide the first intervention session.

### Intervention

The intervention was delivered by the RESPOND clinician (one person from a team of 3 physiotherapists, 2 occupational therapists, 1 nurse, and 1 dietitian) in a face-to-face session in the participant’s home and then via telephone during the 6 months after recruitment. All clinicians attended a 2-day face-face study-specific training session on falls, patient-centred care, the RESPOND program, motivational interviewing, and behaviour change strategies. This was facilitated by the lead clinician, who had attended a motivational interviewing course. Training was followed with a subsequent shadowing session, regular check-ins by the lead clinician, and competency reviews. A standard operating procedures (SOPs) manual guided consistent delivery of program content and intended delivery style, across sites. Clinicians were also experienced in falls prevention assessment and management, including completing home safety assessments and prescribing falls prevention exercises. At the face-to-face session, the RESPOND clinician discussed the falls risk assessment findings with the participant, including their falls risk status, and identified falls risk factors and potential management strategies. They provided participants with the education leaflets for the four RESPOND modules (available from authors on request): (1) better strength, (2) better vision, (3) better sleep, and (4) better bones. These modules were evidence based and provided information on the management of risk factors (e.g., exercise, vision testing and revision of glasses prescription, home safety assessment and modification, withdrawal of sedatives, and vitamin D testing and supplementation), presented as positive health messages. Based on risk factors identified, participants were encouraged, through motivational interviewing, to choose one or more of the four modules that appealed to them and develop personalised goals and action plans for each one. As this intervention used a patient-centred approach, patients were actively involved in their care and decisions regarding their treatment. As such, participants were free to choose the modules they perceived most relevant to them. Recommendations provided by ED staff were also reviewed and discussed with participants. Barriers to the patient achieving their action plans were identified by the clinician and through motivational interviewing these were resolved, when possible. Throughout the session, positively framed messages were adopted by the clinician.

Within two weeks of the face-to-face session, the clinician telephoned the participant to review their progress with goals and action plan. Using motivational interviewing techniques, they provided encouragement and affirmation to implement their action plan, support to troubleshoot issues hampering participants’ ability to implement their plan, and additional education on risk factor management. Subsequent telephone calls were conducted during the 6 months of the active intervention phase, at times nominated by the participant. It was expected that participants would receive a minimum of two follow-up phone calls. Intervention details according to the CONSORT extension Template for Intervention Description and Replication (TIDieR) guidelines have been published elsewhere [20]. S1 Table provides three examples of the participants’ journey on the RESPOND program.

### The comparator

Control participants received the same baseline assessment, letter to usual care medical practitioner, and standard care as arranged/initiated by ED staff (e.g., investigations, multidisciplinary assessment, referral to specialists and falls prevention services, post-discharge nurse telephone contact) and their healthcare providers as RESPOND participants. No usual care treatments were withheld from the control group.

### Outcomes

Primary outcomes were falls and fall injuries per person-year over the 12-month study period. A fall was defined as above. A fall injury was defined as any physical harm resulting from a fall (including fractures, dislocations, sprain, skin tears, and bruising) reported by study participants [30]. Falls may result in multiple injuries. As such, data on injurious falls (falls with at least one injury) were also recorded. Secondary outcomes included ED re-presentations, hospitalisations, fractures (confirmed by radiological investigation), falls risk, falls efficacy, quality of life, and deaths per person-year over the 12-month study period.

Fall and hospitalisation outcomes were collected via postal-returned monthly calendars and telephone calls. All participants were asked to complete calendars daily, recording information about outcomes using tick boxes, and received a monthly telephone follow-up call from an outcome assessor blinded to group allocation to verify information recorded. When an ED presentation or hospital admission was reported, this was verified with participating hospital administrative records. Deaths were identified in hospital administrative data sets or as notified by family or caregiver at monthly follow-up. Falls risk status (FROP-Com risk score and category) [25,26], falls self-efficacy (FES-I) [15], and health-related quality of life (EQ-5D-5L) [28,29] were collected by the blinded outcome assessors at 6- and 12-month follow-up assessments, conducted in the participants’ home using standardised procedures.

### Statistical analysis

An a priori sample size calculation was undertaken [20]. As falls are more frequent than fall injuries, the sample size calculation was based on fall injuries. We identified that we would have 80% power to detect a rate ratio of 0.70 for fall injuries between intervention and control groups at the 5% significance level if 264 participants per group were recruited (assuming a control fall injury rate of 1.01 per person-year [13] and an overdispersion parameter of 1.5), allowing for 20% loss to follow-up [13]. This sample size would provide 80% power to detect a rate ratio of 0.70 for ED re-presentations between intervention and control groups at the 5% significance level, assuming a control re-presentation rate of 0.71 [13].

Outcome analyses were undertaken on an intention-to-treat basis using all available data for each patient. All participants who completed a baseline assessment and provided at least one monthly calendar or telephone call were included in the primary outcome analysis. The exposure time was calculated for each participant from the date of recruitment to 365 days or the last date of calendar data recorded if follow-up was incomplete. Rates were calculated per person-year of exposure time and compared between groups using negative binomial regression models, including a variable for adjustment by site and an offset for exposure time, and with robust standard errors to account for additional or differential overdispersion between groups.

All participants who completed baseline and 12-month assessments were included in the analyses of falls risk, quality of life, and falls efficacy. Mean differences in falls risk, quality of life, and falls efficacy scores between groups at the 12-month follow-up were evaluated using an independent *t* test. Differences between groups in the proportion of people classified as high falls risk on the FROP-Com and those reporting a problem with mobility, self-care, usual activities, pain or discomfort, or anxiety and depression on the EQ-5D-5L were evaluated using a binomial test of proportions. A significance level of *P* < 0.05 was used to indicate statistical significance. All analyses were undertaken using Stata v14.

## Results

### Participants

**Fig 2:**
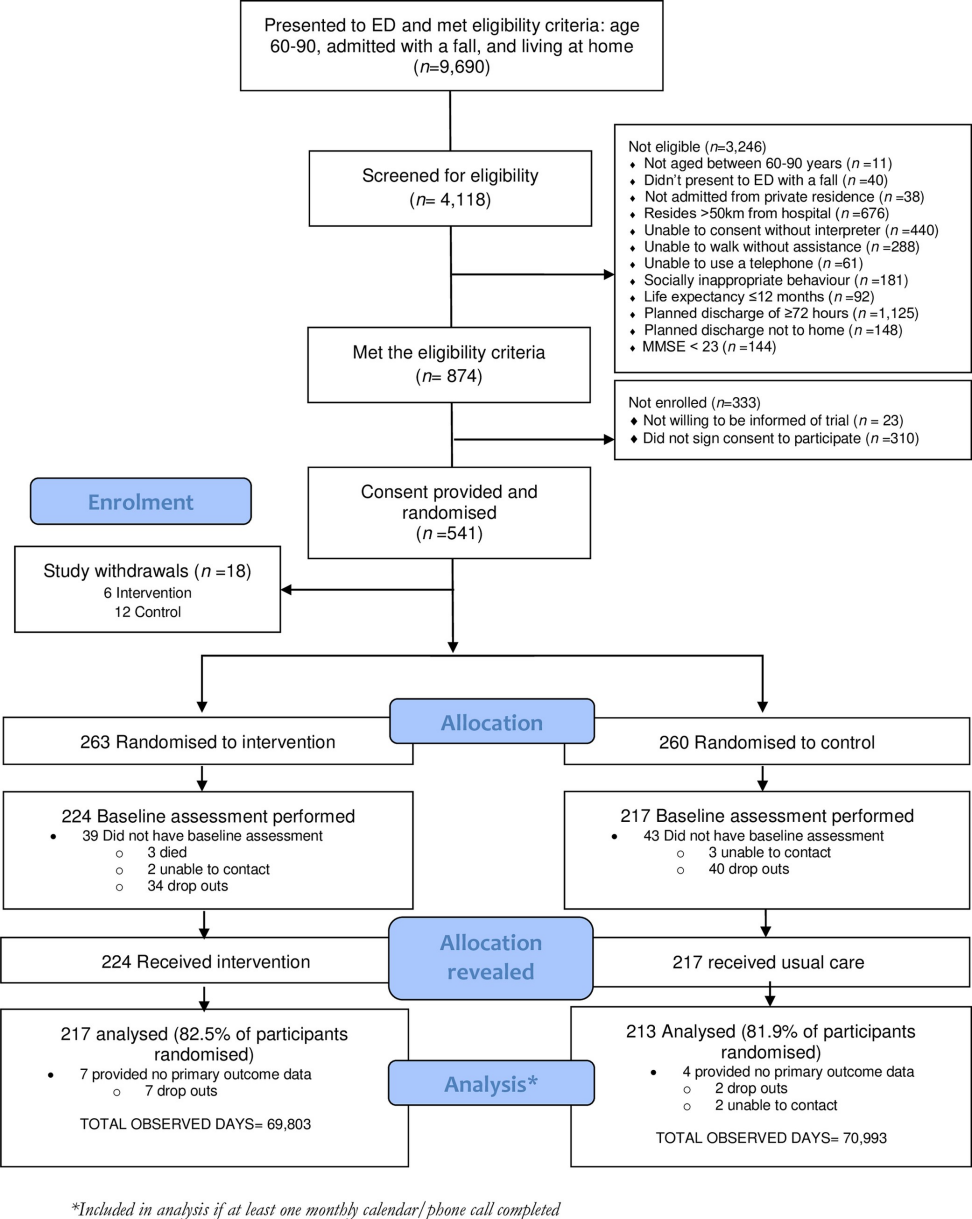
Participant flow through the RESPOND RCT. ED, emergency department; MMSE, mini-mental state examination; RCT, randomised controlled trial.

Administrative data from participating hospitals indicated that during the recruitment period, 9,690 people aged 60 to 90 years presented to the two EDs with a fall and were discharged home. A total of 541 participants were recruited to the study (289 from The Alfred Hospital and 252 from Royal Perth Hospital). Fig 2 outlines the flow of participants through the study. Of those recruited, 430 (79.5%) provided at least one monthly calendar and were included in the primary outcome analysis. The most common reason for participants exiting the study prior to the 12-month follow-up was a complex health situation (*n* = 65, 41.9%) (S2 Table).

Demographics of participants were similar for intervention and control groups (Table 1), with slightly more females than males and a mean age of 73. Two out of five lived alone, and just over 40% had experienced one or more falls in the last 12 months. Three out of five reported taking four or more medications at the baseline assessment. A total of 224 (85.2%) intervention participants received the initial face-to-face intervention session in their home following their baseline assessment. Of the 224 intervention participants, 217 (96.9%) received at least one follow-up phone call (median number of calls = 6; range: 0–19). The most common risk factor selected by participants to address was poor balance and/or loss of strength (*n* = 204, 91.1% of participants who received intervention), followed by poor bone health (*n* = 148, 66.1%), poor sleep (*n* = 81, 36.2%), and poor vision (*n* = 72, 32.1%).

**Table 1:** Participant characteristics at baseline.

Characteristics	Total cohort		Participants included in primary outcome analysis^a^	
	Intervention, *n* = 263	Control, *n* = 260	Intervention, *n* = 217	Control, *n* = 213
Female, *n* (%)	132 (50.2)	156 (60.0)	109 (50.2)	126 (59.2)
Age, mean (SD)	73 (8.4)	73 (8.6)	73 (8.3)	72 (8.3)
Age group, *n* (%)				
60–69	107 (40.7)	111 (42.7)	93 (42.9)	93 (43.7)
70–79	89 (33.8)	83 (31.9)	72 (33.2)	68 (31.9)
80–90	67 (25.5)	66 (25.4)	52 (24.0)	52 (24.4)
Hours of index admission, median (IQR)	17.9 (4.3–49.1)	20.1 (3.6–49.2)	15.8 (4–42.2)	18.5 (3.5–49.2)
**Baseline assessment**	***n* = 224**	***n* = 217**	***n* = 217**	***n* = 213**
Lives alone, *n* (%)	93 (41.5)	94 (43.3)	88 (40.6)	91 (42.7)
Employed, *n* (%)	50 (22.3)	40 (18.4)	48 (22.1)	37 (17.4)
**FROP-Com**^**b**^				
Reported ≥1 fall in last 12 months (excluding index fall)	89 (39.7)	93 (42.9)	83 (38.2)	91 (42.7)
Most severe injury sustained from a fall in the last 12 months, *n* (%)				
No injuries	4 (1.8)	5 (2.3)	4 (1.8)	5 (2.3)
Minor injury—no medical attention	5 (2.2)	6 (2.8)	5 (2.3)	6 (2.8)
Minor injury—medical attention	121 (54.0)	92 (42.4)	117 (53.9)	91 (42.7)
Severe injury	94 (42.0)	114 (52.5)	91 (41.9)	111 (52.1)
Sedative medication	37 (16.5)	29 (13.4)	36 (16.6)	29 (13.6)
Antidepressant medication	52 (23.2)	51 (23.5)	51 (23.9)	49 (22.6)
Anti-epileptic medications	15 (6.9)	10 (4.5)	15 (7.0)	10 (4.6)
Central analgesic medications	45 (20.1)	52 (24.0)	44 (20.3)	52 (24.4)
Number of prescription medications, *n* (%)				
No medications	16 (7.1)	20 (9.2)	16 (7.4)	20 (9.4)
1–2 medications	45 (20.1)	37 (17.1)	42 (19.4)	36 (16.9)
3 medications	26 (11.6)	33 (15.2)	25 (11.5)	32 (15.0)
4 or more medications	137 (61.2)	127 (58.5)	134 (61.8)	125 (58.7)
Medical conditions reported by participants, *n* (%)				
Arthritis	86 (38.4)	103 (47.5)	83 (38.2)	102 (47.9)
Cardiac condition	72 (32.1)	68 (31.3)	68 (31.3)	66 (31.0)
Respiratory condition	52 (23.2)	44 (20.3)	50 (23.0)	44 (20.7)
Diabetes	45 (20.1)	38 (17.5)	44 (20.3)	37 (17.4)
Osteoporosis	36 (16.1)	34 (15.7)	33 (15.2)	34 (16.0)
Stroke	18 (8.0)	23 (10.6)	18 (8.3)	22 (10.3)
Other	73 (32.6)	71 (32.7)	71 (32.7)	70 (32.9)
Number of comorbidities, *n* (%)				
None	53 (23.7)	44 (20.3)	51 (23.5)	42 (19.7)
1	55 (24.6)	53 (24.4)	55 (25.3)	53 (24.9)
2	53 (23.7)	56 (25.8)	52 (24.0)	55 (25.8)
≥3	63 (28.1)	64 (29.5)	59 (27.2)	63 (29.6)
Vision issues, *n* (%)	115 (51.3)	109 (50.2)	110 (50.7)	108 (50.7)
Total score (0–60) (mean, SD)	16.4 (6.1)	16.6 (5.6)	16.4 (6.1)	16.6 (5.6)
Mild, *n* (%)	54 (24.1)	41 (18.9)	53 (24.4)	40 (18.8)
Moderate, *n* (%)	90 (40.2)	107 (49.3)	86 (39.6)	105 (49.3)
High, *n* (%)	80 (35.7)	69 (31.8)	78 (35.9)	68 (31.9)
**EQ-5D-5L**^**c**^				
Overall health state (0–100) (mean, SD)	71.2 (18.9)	71.5 (18.3)	71.5 (18.6)	71.3 (18.3)
Utility score (0–1) (mean, SD)	0.6 (0.3)	0.6 (0.3)	0.6 (0.3)	0.6 (0.3)
Reported problem				
Mobility, *n* (%)	133 (59.3)	116 (53.5)	127 (58.5)	116 (54.4)
Self-care, *n* (%)	89 (39.7)	83 (38.2)	87 (40.1)	83 (39.0)
Usual activity, *n* (%)	138 (61.6)	143 (65.9)	133 (61.3)	142 (66.7)
Pain/discomfort, *n* (%)	168 (75.0)	175 (80.6)	163 (75.1)	173 (81.2)
Anxiety/depression, *n* (%)	102 (45.5)	105 (48.4)	100 (46.1)	103 (48.4)
**Short FES-I**^**d**^				
Total score (0–28) (mean, SD)	11.6 (4.8)	11.6 (5.1)	11.6 (4.8)	11.7 (5.1)

^a^At least one monthly calendar/phone call completed.

^b^Higher scores on the FROP-Com indicate increased falls risk.

^c^Higher scores on the EQ-5D-5L indicate better overall health state.

^d^Higher scores on the FES-I indicate increased fear of falling.

Abbreviations: EQ-5D-5L, EuroQol five dimensions questionnaire; FROP-Com, Falls Risk for Older People in the Community; IQR, interquartile range; SD, standard deviation; Short FES-I, Falls Efficacy Scale–International (Short version).

### Outcomes

Over the 12-month study period, there were 575 falls, 475 fall injuries, 284 injurious falls, and 33 fractures recorded for 206 participants (Table 2). There were 295 self-reported ED re-presentations in 154 people and 399 hospital admissions in 190 people. Of these, a high proportion (32.9% ED re-presentations [*n* = 97] and 46.4% hospital admissions [*n* = 185]) were reported by participants to have occurred at nonparticipating hospitals. A total of 80 ED re-presentations (27.1%) and 83 hospitalisations (20.8%) were reported to be related to a fall.

**Table 2:** Description of outcome events over the 12-month follow-up.

Outcome events	Intervention	Control	All
	(*n* = 217)	(*n* = 213)	(*n* = 430)
Observed days	69,803	70,993	140,796
Number of falls, *n*	220	355	575
Number of fallers, *n* (%)	100 (46.1)	106 (49.8)	206 (47.9)
Multiple fallers, *n* (%)			
0 fall, *n* (%)	117 (53.9)	107 (50.2)	224 (52.1)
1 fall, *n* (%)	53 (24.4)	48 (22.5)	101 (23.5)
2 falls, *n* (%)	19 (8.8)	24 (11.3)	43 (10.0)
≥3 falls, *n* (%)	28 (12.9	34 (15.9)	62 (14.4)
Number of injurious falls^a^, *n* (%)	112 (50.9)	172 (48.5)	284 (49.4)
Number of fall injuries^b^, *n*	206	269	475
Bruise, *n* (%)	103 (50.0)	159 (59.1)	262 (55.2)
Skin injury^c^, *n* (%)	63 (30.6)	67 (24.9)	130 (27.4)
Sprain or strain, *n* (%)	18 (8.7)	7 (2.6)	25 (5.3)
Fractures (all), *n* (%)	10 (4.9)	23 (8.6)	33 (6.9)
NOF, *n* (%)	0 (0.0)	0 (0.0)	0 (0.0)
Pelvis, *n* (%)	1 (10.0)	1 (4.3)	2 (6.1)
Peripheral, *n* (%)	6 (60.0)	19 (82.6)	25 (75.8)
Rib, *n* (%)	3 (30.0)	1 (4.3)	4 (12.1)
Vertebrae, *n* (%)	0 (0.0)	1 (4.3)	1 (3.0)
Other injuries, *n* (%)	12 (5.8)	13 (4.8)	25 (5.3)
Deaths, *n* (%)	2 (1.0)	1 (0.5)	3 (0.7)
ED presentations (all cause), *n*	141	154	295
Hospitalisation (all cause), *n*	173	226	399

^a^Injurious falls is a count of all falls resulting in injury.

^b^Fall injuries is a count of all injuries resulting from falls.

^c^Graze, laceration, skin tear.

Peripheral fractures were defined as non-vertebral, -skull, -face, or -pelvic fractures.

Abbreviations: ED, emergency department; NOF, neck of femur.

Fewer falls (incidence rate ratio [IRR]: 0.65, 0.43–0.99; *P* = 0.042) were observed in the RESPOND group compared with the control (Table 3). There was no difference in fall injuries (IRR 0.81, 0.51–1.29, *P* = 0.374) but a nonsignificant reduction in injurious falls (IRR 0.66, 0.43–1.03, *P* = 0.069). Fewer fractures (IRR 0.37, 0.15–0.91, *P* = 0.030) were also observed in the RESPOND group compared with the control. There were no significant differences in ED re-presentation (IRR 0.92, 0.64–1.32, *P* = 0.653) or hospitalisations (IRR 0.78, 0.55–1.10, *P* = 0.152) between groups. Analyses were unadjusted as there was no difference in age or cognitive status between groups in the primary analysis cohort, and no significant site effect was detected in the analyses. Consistent results were observed in secondary analyses adjusting for sex, with fewer falls observed in the RESPOND group (IRR 0.65, 0.43–0.97, *P* = 0.034) but no difference in fall injuries between groups (IRR 0.87, 0.53–1.34, *P* = 0.530). When undertaking analyses accounting for clustering of patient outcomes by treating clinician using random effects models [31], these models failed to converge due to estimating a zero variance component, indicating that there was no clustering of outcomes. A sensitivity analysis was also performed, in which we truncated the number of falls for those who had 8 or more falls to the value of 8 (S3 Table), to explore if a small number of frequent fallers was unduly influencing the results. This analysis removed 168 falls in seven people and resulted in a nonsignificant reduction in falls (IRR 0.84, 0.63–1.12, *P* = 0.249).

**Table 3:** Outcomes at 12 months follow-up compared between the intervention and the control groups.

Outcomes	Rates per person-years		Rate ratio	*P*
	Intervention	Control		value
	(*n* = 217)	(*n* = 213)	(95% CI)	
**Primary endpoints**				
Falls	1.15 (1.00–1.31)	1.83 (1.64–2.03)	0.65 (0.43–0.99)	0.042
Fall injuries	1.08 (0.94–1.23)	1.38 (1.22–1.56)	0.81 (0.51–1.29)	0.374
**Secondary endpoints**				
Fractures	0.05 (0.03–0.10)	0.12 (0.07–0.18)	0.37 (0.15–0.91)	0.030
ED presentations	0.74 (0.62–0.87)	0.79 (0.67–0.93)	0.92 (0.64–1.32)	0.653
Hospitalisations	0.90 (0.77–1.05)	1.16 (1.02–1.16)	0.78 (0.55–1.10)	0.152
Deaths	0.01 (0.00–0.04)	0.01 (0.00–0.03)	NA^a^	

^a^Not applicable, as there were too few events.

Abbreviation: ED, emergency department.

There were no significant differences in any of the remaining secondary outcomes between groups. Detailed results relating to falls risk, quality of life, and falls efficacy scores are reported in S4 Table. No adverse events or unintended harm were reported to the research team for any participant during the study period.

## Discussion

In this study, RESPOND, a telephone-based, patient-centred intervention led to a significant reduction in falls but not fall injuries in older people who presented to the ED with a fall. There was also an apparent reduction in fractures, but no change in hospitalisations, ED presentations, death, fall risk, falls efficacy, or quality of life. RESPOND adopted some different delivery approaches compared with traditional falls prevention interventions. The use of telephone-delivered goal setting, motivational interviewing, and coaching using positive health messages aimed to provide person-centred care in evidence-based fall prevention strategies. There were only four risk factors targeted in RESPOND, in contrast to open targeting of multiple identified falls risk factors in prior studies. This targeted approach was a deliberate strategy to optimise implementation and uptake.

This study used a rigorous RCT design across two sites, with allocation concealment, collection of data from multiple sources, blinded outcome assessment, and intention-to-treat analysis. However, this study has some limitations. There was a high number of dropouts from the study. This was not unexpected and is likely to reflect the complex conditions and social circumstances of many older people who present to the ED [32]. Indeed, the sample size calculation was based on an expected 20% loss to follow-up. Importantly, nearly 80% (*n* = 74) of the total trial dropouts (*n* = 93) occurred prior to baseline assessment and revealing of group allocation. Therefore, the dropouts are unlikely to have biased results (S5 Table). However, this highlights the challenges of undertaking a trial in this setting, in which individuals have complex health profiles that may impact on their interest and capacity to participate in clinical research and indeed health interventions. There is a risk of co-intervention in this study. The falls risk status of each participant was communicated to their primary care doctor; this may have prompted them to implement falls prevention strategies. While falls data were collected via multiple sources, falls and injuries may have been underreported across both groups due to recall bias. Concomitantly, those in the intervention group may have subconsciously or consciously been less likely to report falls because of their engagement with the intervention and their own personal efforts and investment in falls prevention, a potential weakness of falls prevention trials in general and not isolated to this study. Finally, whilst we included all participant-reported hospital utilisation events, almost half could not be verified in hospital administrative data, as they reportedly occurred at hospitals other than those participating in the study. However, the proportion of admissions that occurred at nonparticipating hospitals was similar across groups, suggesting low measurement bias. Statistically, the fracture rate reduction was based on a relatively small number of fracture events (*n* = 33 fractures), which leaves open the possibility that results may not be replicable in a larger study with more events.

There have been nine previous RCTs that have examined the effectiveness of multifactorial falls prevention programs in older people who present to the ED with a fall [13,15,33–39]. Of these, only two reported a reduction in falls. The first, ‘Prevention of Falls in the Elderly Trial (PROFET)’ RCT of 397 older people in the UK, achieved a reduction in recurrent falls through a multidisciplinary program [35]. However, when the PROFET intervention was tested in the Netherlands it was found to have no impact on falls [15]. A second UK trial of 313 older people presenting to ED with recurrent falls found a multifactorial intervention reduced falls by 36% [33]. There were some common elements across RESPOND and the interventions tested in these two trials. All utilised assessments to identify risk factors for management and all applied a multifactorial intervention that included exercise, vision correction, and home safety modifications.

The event rates for fall injuries and hospitalisation outcomes were lower than for falls, meaning that there was insufficient power to detect smaller effects than the hypothesised 30% reduction. When the number of falls was truncated in the sensitivity analysis there was a nonsignificant reduction in falls. Whilst it may be possible that this intervention only reduces falls in frequent fallers, the study was not originally designed to test this. This additional hypothesis should be further explored in future studies. Furthermore, the lack of effect on fall injuries and hospitalisation outcomes may highlight that more intensive interventions are required to affect these endpoints, and the broader health issues in this population may have required interventions in addition to those included in RESPOND. Four out of five rehospitalisations were for reasons other than a fall, highlighting the complex health profile of older people attending the ED following a fall. Participants had multiple comorbidities—one in three had a cardiac condition, one in two diabetes or arthritis, one in ten a stroke, and almost two in three were taking four or more prescribed medications. The RESPOND intervention did not specifically target these conditions. On average, participants received one home visit and six phone calls over the 6- month intervention period. This dosage may be too low to modify the complex health issues that may lead to hospital utilisation. In addition, there is likely substantial clinical heterogeneity across the participants [11], which may contribute to a smaller effect size, as observed in this trial.

### Implications for clinicians and policy makers

The key point of difference for RESPOND in comparison with prior studies was ‘how’ the intervention was delivered. By using a person-centred telephone-delivered approach, the program aimed to provide care responsive to individual patient preferences, needs, and values, with an inexpensive and wide-reaching method [40]. Telephone delivery enables broad delivery and scale, and whilst this study used one face-to-face session, future research could test an entirely telephone-based delivery to see if outcomes are maintained. Qualitative studies with older adults have identified that negatively framed falls prevention messages are often perceived as patronising and a threat to independence [41,42]. Positive health messaging was utilised in RESPOND to enhance engagement and participation in program components and may be a key component for clinicians to consider in future falls prevention programs.

### Future research

Further studies that involve a larger sample size would provide information as to whether smaller yet clinically meaningful effects on fall injuries and hospitalisation outcomes exist with the RESPOND intervention [43]. Similar studies should also be conducted to explore the effect on specific subgroups such as frequent fallers. The recruitment processes used in this trial are likely beyond the scope and resources available in most usual care settings that seek to implement the RESPOND program. Therefore, there is also a need for rapid yet cost-effective processes to identify which older fallers presenting to the ED should be prioritised to receive this intervention. Program participation and the associated barriers and facilitators will be explored in greater detail as part of a multilevel program evaluation of RESPOND [44]. In addition, while sociodemographic factors such as social support were not specifically captured by this study, the result that almost one in two participants lived alone highlights that many older people who present to the ED with a fall may lack social support. Prior studies suggest that adequate social support is essential for functional recovery and maintenance of community living [45–47]. The RESPOND program did not aim to improve social support, and this highlights a potential remaining unmet need in many older fallers presenting to the ED. It may be appropriate to consider adapting the RESPOND program to further target social connectedness or social support.

### Conclusion

In this study, the RESPOND program reduced falls and fractures in community-dwelling older people presenting to the ED with a fall, but there was no significant difference in fall injuries. Key points of difference between this intervention and those tested previously are the inclusion of telephone-delivered patient-centred care techniques, motivational interviewing, and positive health messages.

## Supporting information

S1 CONSORT Checklist (DOCX)

S1 Table Case studies of three individual participants (pseudonyms used, actual data collected). (DOCX)

S2 Table Reasons for early study exit by group (excluding deaths), between randomisation and 12-month follow-up. (DOCX)

S3 Table Multiple fallers. (DOCX)

S4 Table Falls risk, health-related quality of life and falls efficacy scores at baseline, and 12-month follow-up. (DOCX)

S5 Table Participant characteristics of study dropouts. (DOCX)

## Abbreviations

CHOICE: Choice of Health Options In prevention of Cardiovascular Events
ED: emergency department
EQ-5D-5L: EuroQol five dimensions questionnaire
FROP-Com: Falls Risk for Older People in the Community
HLQ: Health Literacy Questionnaire
IQR: interquartile range
IRR: incidence rate ratio
MMSE: Mini-Mental State Examination
NOF: neck of femur
PROFET: Prevention of Falls in the Elderly Trial
RCT: randomised controlled trial
SD: standard deviation
Short FES-I: Falls Efficacy Scale–International (Short version)
SOP: standard operating procedure
TIDieR: Template for Intervention Description and Replication
