# Supplementary material for: Evaluation of RESPOND, a patient-centred program to prevent falls in older people presenting to the emergency department with a fall: A randomised controlled trial
Source: PLoS Med. 2019 May 24;16(5):e1002807. doi: 10.1371/journal.pmed.1002807 (PMC6534288; doi:10.1371/journal.pmed.1002807)
Supplement: S1 Table — (DOCX) [file pmed.1002807.s002.docx]

**S1 Table: Case studies of three individual participants (pseudonyms used, actual data collected)**

|  | **Frieda**  Frieda is a 72 year old lady who presented to the ED following a fall at home. She was unpacking her shopping when she tripped and fell forward onto her face. | **Albert**  Albert is an 88 year old man who fell on a bus as it jerked forward. He fell onto his bottom and was take to the ED via ambulance. | **Jan**  Jan is a 65 year old lady who slipped on the floor at home when walking around in socks. She fell onto an outstretched hand, resulting in a fractured wrist. |
| --- | --- | --- | --- |
| **Risk factors and goals at baseline assessment (home visit)** | | | |
| **FROM-COM score** | 21 (high falls risk) | 25 (high falls risk) | 9 (mild falls risk) |
| **Risk Factors as identified on the FROP-COM** | Polypharmacy, more than one co-morbidity, visual, foot and mobility problems (uses a walking stick at home and in the community), footwear problem, difficulty getting to the toilet in time, 1 alcoholic drink over past week, unsafe moving around the house, at risk of losing balance. | Polypharmacy, more than one co-morbidity, visual, foot and mobility problems (Albert uses a gait aid in the community), footwear problem, unsafe moving around the house, home environment does not appear safe, at risk of losing balance. | Footwear problem, change in assistance required for domestic tasks. |
| **History of falls** | 2 falls in the past 12 months | 1 fall in the past 12 months | 1 fall in the past 12 months |
| **Goals** | To build strength and confidence when walking down the street so that she doesn’t have another fall.  To be able to sleep better as she wakes often during the night. | To build confidence with using public transport and get out and about.  To see the eye specialist.  To be able to get less disturbed sleep. | To understand more about how she can increase her bone strength so she is less likely to get a broken bone if she does fall again. |
| **Module(s) selected by participant** | Better sleep  Better strength and balance | Better strength and balance  Better bones  Better eyesight  Better sleep | Better bones |
| **RESPOND intervention** | | | |
| **Number of calls** | 8 coaching calls | 7 coaching calls | 4 coaching calls |
| **Barriers discussed** | Hindered by long term back pain and multiple co-morbidities. Heart condition restricts the amount of physical exercise she is able to do. Energy levels low (stated as due to age). Living alone (some isolation reported), expectations from self and family to cook feasts. | Self-professed procrastinator, often not following up on things he agreed to do. Also stated didn’t want to rock the boat at his age (“I’m too old to do something new”). | Unable to drive, carer for husband, prioritises his needs over her own. |
| **How motivational interviewing was used** | Sleeps well on some nights and terribly on others – discussed factors that may be contributing to this and reinforced sleep hygiene strategies each call.  Worries about driving to appointments so sleeps poorly the night before. Strategies discuss around managing this – ended up getting taxis to appointments which improved all aspects.  Graded exercise programme discussed and adapted for limited abilities and physical restrictions. | Poor sleeping habits due to pain from injuries. Discussed sleep hygiene strategies and other ways of dealing with pain – mindset changes.  Was being monitored yearly through the eye hospital regarding need for another. Eyesight causing difficulties mobility.  Discussion around how pain was limiting and impacting on his independence. Identification of small steps he ‘could’ achieve. Conversations at times around consequences of inaction, celebration of changes made throughout the 6 months.  Discussion around how sunlight can increase bone health. | Discussion about importance of putting self-first, at times to be able to provide ongoing care for husband. Identified the importance of understanding bone density and value of a DEXA scan and safe sun exposure on a daily basis. |
| **Outcomes** | | | |
| **Goals achieved** | Progressed to using walking stick mainly in the community and not at home.  Sleep still interrupted at times, but less anxious about this and has good strategies in place when it happens. | Medications reduced from 3 to 1 and reduced use of benzodiazepines.  Progressed to walking without a walking stick again in the community.  Doing parts of the home exercise programme intermittently. More confident to go into the community. Less anxiety about living with pain. Beginning to spend more time outside in the daylight each day. | No additional falls during 12 months of study. Had a DEXA scan and discussing results with GP. |
| **Number of falls** | 0 | 0 | 0 |
| **Number of hospitalisations during 12 month follow-up** | 2 admissions unrelated to falls | 1 admission unrelated to falls | No admissions |

FROP-Com Falls Risk for Older People in the Community, higher scores on the FROP-Com indicate increased falls risk

ED= Emergency Department

GP= General practitioner

DEXA= dual-energy x-ray absorptiometry
