# Supplementary material for: Evaluation of RESPOND, a patient-centred program to prevent falls in older people presenting to the emergency department with a fall: A randomised controlled trial
Source: PLoS Med. 2019 May 24;16(5):e1002807. doi: 10.1371/journal.pmed.1002807 (PMC6534288; doi:10.1371/journal.pmed.1002807)
Supplement: S2 Table — (DOCX) [file pmed.1002807.s003.docx]

**S2 Table: Reasons for early study exit by group (excluding deaths), between randomisation and 12-month follow up**

|  | **Control** | | **Intervention** | |
| --- | --- | --- | --- | --- |
|  | N=75 | | N=80 | |
| Complex health situation, n (%) | 32 | (42.7) | 33 | (41.3) |
| Complex social situation, n (%) | 8 | (10.7) | 7 | (8.8) |
| Insufficient time, n (%) | 9 | (12.0) | 11 | (13.8) |
| Not willing to have home visit, n (%) | 4 | (5.3) | 4 | (5.0) |
| High participation burden, n (%) | 10 | (13.3) | 9 | (11.3) |
| Lack of perceived benefit, n (%) | 6 | (8.0) | 6 | (7.5) |
| No reason given, n (%) | 6 | (8.0) | 10 | (12.5) |
