# Supplementary material for: Evaluation of RESPOND, a patient-centred program to prevent falls in older people presenting to the emergency department with a fall: A randomised controlled trial
Source: PLoS Med. 2019 May 24;16(5):e1002807. doi: 10.1371/journal.pmed.1002807 (PMC6534288; doi:10.1371/journal.pmed.1002807)
Supplement: S3 Table — (DOCX) [file pmed.1002807.s004.docx]

**S3 Table Multiple fallers**

|  | **Intervention** | **Control** | **All** |
| --- | --- | --- | --- |
|  | **(n=217)** | **(n=213)** | **n=(430)** |
| Number of falls per participant, n  0  1  2  3  4  5  6  7  8  13  14  20  24  25  31  41 | 117  53  19  13  9  1  1  1  2  0  0  1  0  0  0  0 | 107  48  24  14  7  4  1  1  1  1  1  0  1  1  1  1 | 224  101  43  27  16  5  2  2  3  1  1  1  1  1  1  1 |
