# Supplementary material for: Evaluation of RESPOND, a patient-centred program to prevent falls in older people presenting to the emergency department with a fall: A randomised controlled trial
Source: PLoS Med. 2019 May 24;16(5):e1002807. doi: 10.1371/journal.pmed.1002807 (PMC6534288; doi:10.1371/journal.pmed.1002807)
Supplement: S4 Table — (DOCX) [file pmed.1002807.s005.docx]

**S4 Table: Falls risk, health related quality of life and falls efficacy scores at baseline and 12-month follow-up**

|  | **Baseline** | | **12 month follow-up** | |  | |  |  |
| --- | --- | --- | --- | --- | --- | --- | --- | --- |
|  | **Intervention** | **Control** | **Intervention** | **Control** | **Difference**  **(95% CI)** | **P value** | |  |
| **FROP-Com** | *n=224* | *n=217* | *n=164* | *n=178* |  |  | |  |
| Total score (0-60) (mean, SD) | 16.4 (6.1) | 16.6 (5.6) | 13.8 (5.9) | 14.3 (5.6) | -0.48 (-1.70 to 0.74) | 0.439 | |  |
| Mild, n (%) | 54 (24.1) | 41 (18.9) | 67 (40.9) | 62 (34.8) | 0.06 (-0.04 to 0.16) | 0.251 | |  |
| Moderate, n (%) | 90 (40.2) | 107 (49.3) | 69 (42.1) | 78 (43.8) | -0.02 (-0.12 to 0.88) | 0.744 | |  |
| High, n (%) | 80 (35.7) | 69 (31.8) | 28 (17.1) | 38 (21.3) | -0.04 (-0.13 to 0.04) | 0.317 | |  |
| **EQ-5D-5L** | *n=224* | *n=217* | *n=171* | *n=182* |  |  | |  |
| Overall health state (0-100) (mean, SD) | 71.2 (18.9) | 71.5 (18.3) | 79.2 (15.2) | 76.9 (16.9) | 2.25 (-1.13 to 5.64) | 0.190 | |  |
| Utility score (0-1) (mean, SD) | 0.6 (0.3) | 0.6 (0.3) | 0.8 (0.2) | 0.8 (0.2) | 0.03 (-0.02 to 0.08) | 0.231 | |  |
| Reported problem |  |  |  |  |  |  | |  |
| Mobility, n (%) | 133 (59.3) | 116 (53.5) | 67 (39.2) | 79 (43.4) | -0.04 (-0.14 to 0.06) | 0.421 | |  |
| Self-care, n (%) | 89 (39.7) | 83 (38.2) | 23 (13.5) | 32 (17.6) | -0.04 (-0.12 to 0.03) | 0.285 | |  |
| Usual activity, n (%) | 138 (61.6) | 143 (65.9) | 54 (31.6) | 74 (40.7) | -0.09 (-0.19 to -0.008) | 0.076 | |  |
| Pain/discomfort, n (%) | 168 (75.0) | 175 (80.6) | 107 (62.6) | 126 (69.2) | -0.07 (-0.17 to 0.03) | 0.187 | |  |
| Anxiety/depression, n (%) | 102 (45.5) | 105 (48.4) | 50 (29.2) | 59 (32.4) | -0.03 (-0.13 to 0.06) | 0.518 | |  |
| **Short FES-I** | *n=224* | *n=217* | *n=171* | *n=182* |  |  | |  |
| Total score (0-28) (mean, SD) | 11.6 (4.8) | 11.6 (5.1) | 9.9 (3.6) | 10.6 (6.7) | -0.70 (-1.84 to 0.43) | 0.224 | |  |

SD Standard Deviation

FROP-Com Falls Risk for Older People in the Community

EQ-5D-5L EuroQol five dimensions questionnaire

Short FES-I Falls Efficacy Scale – International (Short version)

NB: some participants returned the FES and EQ5-D via mail but did not participate in a face-face 12-month follow up, therefore did not complete a 12-month FROP-Com
