# Supplementary material for: Evaluation of RESPOND, a patient-centred program to prevent falls in older people presenting to the emergency department with a fall: A randomised controlled trial
Source: PLoS Med. 2019 May 24;16(5):e1002807. doi: 10.1371/journal.pmed.1002807 (PMC6534288; doi:10.1371/journal.pmed.1002807)
Supplement: S5 Table — (DOCX) [file pmed.1002807.s006.docx]

**S5 Table: Participant characteristics of study drop outs**

|  | **Total cohort** | |
| --- | --- | --- |
|  | **Participants included in primary analysis**  **n=430** | **Study drop outs**  **n=93** |
| Female, n (%) | 235 (54.7) | 53 (57.0) |
| Age, mean (SD)^b^ | 73 (8.3) | 75 (9.2) |
| Age group, n (%) |  |  |
| 60-69 | 186 (43.3) | 32 (34.4) |
| 70-79 | 140 (32.6) | 32 (34.4) |
| 80-90 | 104 (24.9) | 29 (31.2) |
| Hours of index admission, median (IQR) | 17.3 (3.8, 46.9) | 23.7 (5.3, 51.0) |
| **Baseline assessment** | **n=430** | **n=11** |
| Lives alone, n (%) | 179 (41.6) | 8 (72.7) |
| Employed, n (%) | 85 (19.8) | 5 (45.5) |
| **FROP-Com^d^** |  |  |
| Reported ≥ 1 fall in last 12 months (excluding index fall) | 174 (40.5) | 8 (72.7) |
| Number of prescription medications, n (%)^c^ |  |  |
| No medications | 36 (8.4) | 0 (0.0) |
| 1-2 medications | 78 (18.1) | 4 (36.4) |
| 3 medications | 57 (13.3) | 2 (18.2) |
| 4 or more medications | 259 (60.2) | 5 (45.5) |
| Medical conditions reported by participants, n (%)^c^ |  |  |
| Arthritis | 185 (43.0) | 4 (36.4) |
| Cardiac condition | 134 (31.2) | 6 (54.5) |
| Respiratory condition | 94 (21.9) | 2 (18.2) |
| Diabetes | 81 (18.8) | 2 (18.2) |
| Osteoporosis | 67 (15.6) | 3 (27.3) |
| Stroke | 40 (9.3) | 1 (9.1) |
| Number of comorbidities, n (%)^c^ |  |  |
| None | 93 (21.6) | 4 (36.4) |
| 1 | 108 (25.1) | 0 (0.0) |
| 2 | 107 (24.9) | 2 (18.2) |
| ≥ 3 | 122 (28.4) | 5 (45.5) |
| Vision issues | 218 (50.7) | 6 (54.5) |
| Total score (0-60) (mean, SD) **^d^** | 16.5 (5.9) | 16.1 (6.1) |
| Mild, n (%) | 93 (21.6) | 2 (18.2) |
| Moderate, n (%) | 191 (44.4) | 6 (54.5) |
| High, n (%) | 146 (34.0) | 3 (27.3) |
| **EQ-5D-5L^e^** |  |  |
| Overall health state (0-100) (mean, SD) | 71.4 (18.4) | 68.9 (25.5) |
| Utility score (0-1) (mean, SD) | 0.6 (0.3) | 0.7 (0.2) |
| **Short FES-I^f^** |  |  |
| Total score (0-28) (mean, SD) | 11.6 (4.9) | 9.2 (3.2) |

a At least one monthly calendar/phone call completed

b SD Standard Deviation

c Reported by participants as part of the FROP-Com assessment

d Higher scores on the FROP-Com indicate increased falls risk

e Higher scores on the EQ-5D-5L indicate better overall health state

f Higher scores on the FES-I indicate increased fear of falling
